# *CEBPA* bZIP in-frame mutations in acute myeloid leukemia: prognostic and therapeutic implications

**DOI:** 10.1038/s41408-024-01042-6

**Published:** 2024-04-09

**Authors:** Fenghong Zhang, Zhen Shen, Jundan Xie, Jingren Zhang, Qian Wu, Rui Jiang, Xiangyu Zhao, Xiaofei Yang, Suning Chen

**Affiliations:** 1 National Clinical Research Center for Hematologic Diseases, Jiangsu Institute of Hematology, The First Affiliated Hospital of Soochow University, Suzhou, People’s Republic of China; 2 Institute of Blood and Marrow Transplantation, Collaborative Innovation Center of Hematology, Soochow University, Suzhou, China

**Keywords:** Acute myeloid leukaemia, Diseases

**TO THE EDITOR:**

The transcription factor CCAAT/enhancer-binding protein-alpha(*CEBPA*) is a critical mediator of granulocytic differentiation. Mutations of the *CEBPA* gene (*CEBPA*^mut^) occur in 5%–15% of adult AML patients and in-frame mutations within the bZIP domain of *CEBPA* (*CEBPA*^bZIP-inf^) define a distinct entity associated with favorable prognosis in AML patients when treated by conventional chemotherapy [1, 2]. *CEBPA* mutations could activate the BCL2 P2 promoter and induce its expression via interaction with nuclear factor-κB (NF-κB) p50 in hematopoietic cell lines and display a markedly hypermethylated profile by multi-omics analysis in primary leukemia cells [3–5]. Meanwhile, venetoclax plus hypomethylating agents (VEN + HMA) were efficient in AML patients with specific molecular profiles (such as *NPM1*, *IDH2*, etc.) [6, 7]. However, relevant data related to the role of VEN + HMA regimens in *CEBPA*^bZIP-inf^ AML patients is limited. Therefore, in the current study, we retrospectively analyzed the clinical features, co-mutational spectrum, and prognostic role of *CEBPA* mutations, particularly *CEBPA*^bZIP-inf^ mutations, in 996 newly diagnosed adult AML patients inducted with chemo-free regimens or chemotherapy between 2016–2022 at the First Affiliated Hospital of Soochow University in China.

A high frequency of *CEBPA* mutations (17.8%,177/996) and *CEBPA*^bZIP-inf^ cases (13.6%,135/996) were identified in our analysis (Fig. S1). *CEBPA*^bZIP-inf^ patients were diagnosed at a younger age, higher hemoglobin counts, lower platelet counts, more likely to have intermediate cytogenetics (normal karyotype) and less number of co-mutations compared with other *CEBPA* mutated (*CEBPA*^other-mut^) and *CEBPA*^wt^ AML patients (Table S1 and Fig. S2). *CEBPA*^bZIP-inf^ mutations exhibited a higher complete remission/complete remission with incomplete cell recovery (CR/CRi) rate (87.6% vs. 64.1% vs. 47.3%, *P* < 0.001), favorable overall survival (OS) (3-year OS: 91.2% vs. 66.0% vs. 55.3%, *P* < 0.001) and relapse-free survival (RFS) (3-year RFS: 73.8% vs. 52.0% vs. 55.3%, *P* < 0.001) compared with *CEBPA*^other-mut^ and *CEBPA*^wt^ cases in the matched cohort (Fig. S3) (Table S2 and S3).

Among 130 *CEBPA*^bZIP-inf^ patients with available information, 116 cases were inducted by 7 + 3 chemotherapy while 14 patients were by VEN + HMA regimens and consolidated by chemotherapy or hematopoietic stem cell transplantation (HSCT). There was no difference in the baseline and genetic characteristics between the 7 + 3 cohort and the VEN + HMA cohort (Table 1). With a median follow-up time of 22 months, among 13 patients evaluable all attained CR/CRi, 46.2%(6/13) relapsed and 3 patients died in the VEN + HMA group (disease progression, *n* = 1; transplant-related complications, *n* = 1; pneumonia, *n* = 1); simultaneously, 113 of 116 patients attained CR/CRi, 22.1%(25/113) relapsed and 6 cases died in the 7 + 3 group. Intriguingly, the VEN + HMA regimens seemed to show similar CR/CRi rates after one cycle of induction therapy (100.0% vs. 86.2%, *P* = 0.324), lower RFS (1-year RFS: 46.9% vs. 88.9%; *P* < 0.001) and OS (1-year OS: 84.6% vs. 99.1%; *P* < 0.001) than 7 + 3 regimens (Fig. 1A, B). When patients were censored at HSCT, worse 1-year RFS and 1-year OS were also observed in the VEN + HMA cohort (41.5% vs. 83.5%, *P* = 0.003 for RFS; 90.0% vs. 98.4%, *P* = 0.010 for OS) (Fig. 1C, D). In accordance with these findings, the multivariable analysis demonstrated adverse RFS (HR, 2.72; 95% CI: 1.01–7.30; *P* = 0.047) and a trend to dismal OS (HR, 5.14; 95% CI: 0.83–31.60; *P* = 0.078) in VEN + HMA cohort compared to 7 + 3 cohorts (Table S4).

**Table 1:** Baseline characteristics of patients with *CEBPA*^bZIP-inf^ mutations in the 7 + 3 and VEN + HMA cohort.

Variables	7 + 3 (*N* = 116)	VEN + HMA (*N* = 14)	*P*-value
Age in years, median [IQR]	34.5 [29.8,45.0]	40.5 [33.3,49.8]	0.167
Sex (Male), *n* (%)	70 (60.3)	7 (50.0)	0.648
WBC (median [IQR]) × 109/L	16.5 [8.6,68.2]	18.2 [8.3,42.7]	0.943
Hemoglobin, (median [IQR])	102.0 [85.0,115.0]	103.5 [84.8,117.3]	0.834
Platelet, (median [IQR]) × 109/L	26.0 [17.0,49.0]	32.0 [26.3,45.0]	0.554
BM blast(median [IQR]) (%)	56.8 [37.8,70.0]	58.3 [40.7,80.1]	0.555
Co-mutations, *n* (%)			
Activated signaling genes			
* CSF3R*	13 (11.2)	0 (0.0)	0.396
* FLT3-ITD*	15 (12.9)	2 (14.3)	1.000
* KIT*	4 (3.4)	1 (7.1)	1.000
* NRAS*	14 (12.1)	4 (28.6)	0.201
* PTPN11*	6 (5.2)	1 (7.1)	1.000
Transcription factors genes			
* GATA2*	29 (25.0)	5 (35.7)	0.589
* RUNX1*	11 (9.5)	3 (21.4)	0.365
Chromatin modifiers genes			
* ASXL1/2*	4 (3.4)	0 (0.0)	1.000
* BCOR*	2 (1.7)	0 (0.0)	1.000
* EZH2*	12 (10.3)	0 (0.0)	0.439
* SETD2*	6 (5.2)	0 (0.0)	0.844
Tumor suppressors genes			
* WT1*	31 (26.7)	5 (35.7)	0.694
DNA methylation genes			
* DNMT3A*	7 (6.0)	2 (14.3)	0.554
* IDH1/2*	2 (1.7)	1 (7.1)	0.739
* TET2*	12 (10.3)	2 (14.3)	1.000
RNA splicing genes			
* SF3B1/U2AF1/ZRSR2*	2 (1.7)	1 (7.1)	0.739
Cohesin complex genes			
* STAG2*	4 (3.4)	0 (0.0)	1.000
Cytogenetics, *n* (%)	116 (100)	14 (100)	0.739
Favorable risk	0	0	
Intermediate risk	114 (98.3)	13 (92.9)	
Adverse risk	2 (1.7)	1 (7.1)	
Induction response, *n* (%)	116 (100%)	13 (92.9%)	0.311
CR/CRi	100 (86.2)	13 (100.0)	
PR or NR	16 (13.8)	0 (0.0)	
HSCT in CR1, *n* (%)	51 (44.0)	3 (21.4)	0.184

*WBC* white blood cell count, *BM* bone marrow, *CR/CRi* complete remission or CR with incomplete hematologic recovery, *PR* partial response, *NR* no response, *HSCT* hematopoietic stem cell transplantation, *CR1* first complete remission.

**Fig. 1:**
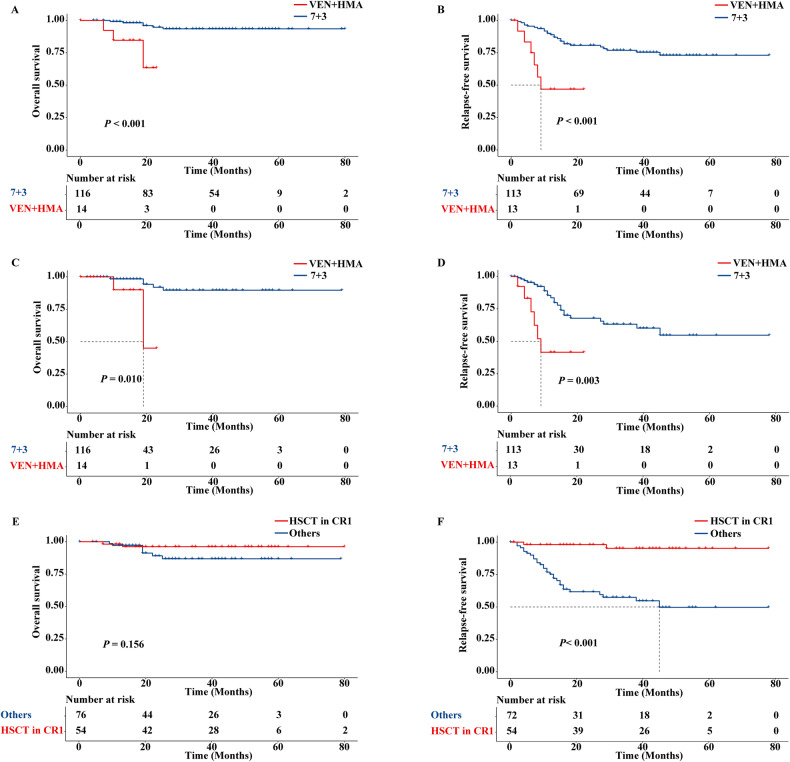
Kaplan-Meier survival curves for overall survival (OS) and relapse-free survival (RFS) in *CEBPA*^bZIP-inf^ patients. **A**, **B** compared survival outcomes among patients inducted with standard 7 + 3 and VEN + HMA regimens; **C**, **D** showed survival outcomes in the standard 7 + 3 and VEN + HMA cohorts with censoring at the time of HSCT; **E**, **F** illustrated survival by consolidation treatment (HSCT in CR1 or not). HSCT hematopoietic stem cell transplantation, CR1 first complete remission.

Totally, 126 *CEBPA*^bZIP-inf^ cases (96.9%) achieved CR/CRi post-induction and 31 of them (24.6%) relapsed, especially in the VEN + HMA cohort with a CR/CRi rate of 100% and relapse rate of 46.2%, which may be different from primary refractory group or persistent remission group and could be classified into “remission but relapse” group. Thus, it’s rather meaningful to explore high-risk indicators (such as co-mutations) for relapse and treatment regimens (such as consolidation) to reduce the relapse rate. With regard to consolidation therapy, 76 patients received cytarabine-based chemotherapy and 54 patients underwent transplantation in CR1 due to Measurable Residual Disease (MRD) positivity, more *PTPN11*, or *FLT3-ITD* mutations. HSCT at CR1 improved RFS (3-year RFS: 95.2% vs. 57.5%; *P* < 0.001) but not OS (3-year OS: 96.2% vs. 86.8%; *P* = 0.156) compared with chemotherapy alone in *CEBPA*^bZIP-inf^ AML patients (Fig. 1E, F), which is consistent with Cox Proportional Hazard Model (HR 0.04, 95% CI: 0.01–0.19; *P* < 0.001 for RFS in multivariable analysis; HR 0.34; 95% CI: 0.07–1.70; *P* = 0.184 for OS in univariable analysis) (Table S4).

On the other hand, overlapping gene mutations may affect the clinical outcome of *CEBPA*^bZIP-inf^ patients. The most frequently co-mutated genes within the *CEBPA*^bZIP-inf^ group were *WT1* (27.7%), *GATA2* (26.2%), *NRAS* (13.8%), *FLT3-ITD* (13.1%) and *RUNX1* (10.8%) (Fig. S2). Notably, *KIT* mutations had a significantly poor impact on RFS (*P* < 0.001) and OS (*P* = 0.002) compared to *KIT* wild type in *CEBPA*^bZIP-inf^ group (Fig. S4), and *CSF3R* mutation showed a worse RFS (*P* = 0.035) by performing landmark analysis after 10 months (crossover) (Fig. S4). Meanwhile, *WT1* and NRAS mutations revealed a tendency toward adverse RFS (*P* = 0.053, *P* = 0.067) and OS (*P* = 0.068, *P* = 0.069) (Fig. S4). Additionally, MDS-related gene mutations (MRs, including *ASXL1*, *BCOR*, *EZH2*, *SF3B1*, *SRSF2*, *STAG2*, *U2AF1*, *ZRSR2*) and *GATA2* aberrations had no effect on survival (Fig. S4).

Vazquez previously found that all 4(100%) *CEBPA*-mutated AML patients achieved CR/CRi but 3(75%) relapsed with a shorter duration of remission (median: 4.65 months) in 19 unfit AML patients treated by venetoclax-based regimens [8]. Similarly, our cohort suggested that VEN + HMA induction exhibited a similar remission rate, worse RFS, and a tendency to poor OS in *CEBPA*^bZIP-inf^ cohort compared with 7 + 3 groups. It seems the treatment depth or clonal evolution of VEN + HMA needs to be further explored. On the side, as earlier studies reported [9, 10], we also demonstrated HSCT in CR1 significantly improved RFS but not OS compared to chemotherapy alone, possibly resulting from a high remission rate of re-induction by chemotherapy after relapse and high transplantation-related mortality. Therefore, HSCT is not recommended in consolidation therapy at first CR in *CEBPA*^bZIP-inf^ AML patients. In other words, conventional chemotherapy is preferred in both induction and consolidation courses among *CEBPA*^bZIP-inf^ AML cases.

The co-occurrence of other genetic mutations has had a controversial prognostic impact in patients with *CEBPA*^mut^ AML by previous studies, generally, mutations of *WT1*, *CSF3R*, *KIT*, *NRAS*, and CCS mutations (mutations in chromatic/DNA modifiers (C), cohesion complex (C), and splicing genes (S)) were associated with adverse prognosis while mutations of *GATA2* were correlated with favorable outcome [9, 11–15]. Similar to these reports, *KIT* mutation was significantly related to inferior RFS and OS in Kaplan-Meier methods (RFS, *P* = 0.002; OS, *P* < 0.001) but not in multivariable analysis (HR 2.45, 95% CI: 0.63–9.55, *P* = 0.197 for RFS; HR 2.63, 95% CI: 0.30-23.04, *P* = 0.381 for OS), a possible explanation maybe the small sample size of *KIT* mutated patients in *CEBPA*^bZIP-inf^ patients. Inferior prognosis in terms of RFS was found in *WT1*(*P* = 0.053), *CSF3R* (*P* = 0.035), and *NRAS* (*P* = 0.069) mutated group while a trend of worse OS without statistical significance was observed (*WT1*, *P* = 0.068; *NRAS*, *P* = 0.067). However, no clinical significance of *GATA2* mutation and MRs were found in the present cohort.

Recently, Georgi divided 1010 *CEBPA*^mut^ adult AML patients into 8 mutational subgroups and refined superior prognosis was associated with in-frame insertions/deletions within bZIP domain of *CEBPA* (*CEBPA* bZIP^InDel^) rather than in-frame mutations within bZIP domain of *CEBPA* (*CEBPA*^bZIP-inf^) by excluding missense mutations within bZIP domain (*CEBPA* bZIP^ms^) [15]. The present *CEBPA*^bZIP-inf^ AML cohort was reanalyzed and 14 *CEBPA* bZIP^ms^ patients were excluded, the prognostic value of VEN + HMA induction and HSCT at CR1 consolidation was consistent with previous results (Fig. S5). Whether truncated mutations in the N-terminus and truncated sites influence the clinical outcome of *CEBPA*-mutated patients were analyzed. No statistical significance of OS (*P* = 0.490) and RFS (*P* = 0.412) were found in *CEBPA* transactivation domain (TAD) frameshift mutated patients and *CEBPA* other mutated patients (Fig. S6). No differences in OS (*P* = 0.240) and RFS (*P* = 0.571) were evaluated in N-terminal truncated mutations spanning the second start codon (Fig. S6).

In conclusion, *CEBPA*^bZIP-inf^ patients exhibited higher CR rates, improved OS and RFS, and benefited from traditional chemotherapy compared with VEN + HMA induction and transplantation consolidation at CR1 in our preliminary study. Furthermore, *CEBPA*^bZIP-inf^ patients with *WT1* and *NRAS* mutation adversely affected the RFS in multivariable analysis. Taken the retrospective nature and limited sample size into consideration, future research efforts aimed at validating our results and elucidating the potential molecular mechanisms are warranted to improve therapeutic strategy in specific types of *CEBPA*^mut^ AML.

### Supplementary information

Supplementary Material

## Data Availability

The data used for this study are not publicly available. Molecular and clinical characteristics are available upon request from the corresponding author.
