# Supplementary material for: CEBPA bZIP in-frame mutations in acute myeloid leukemia: prognostic and therapeutic implications
Source: Blood Cancer J. 2024 Apr 9;14(1):59. doi: 10.1038/s41408-024-01042-6 (PMC11004125; doi:10.1038/s41408-024-01042-6)
Supplement: Supplementary file 1 — Supplementary Material [file 41408_2024_1042_MOESM1_ESM.pdf]

***CEBPA* bZIP in-frame mutations in acute myeloid leukemia: prognostic and therapeutic implications**

Supplemental Information

Supplementary Methods

Patient samples

A total of 996 patients who were diagnosed with AML(non-acute promyelocytic leukemia) in our department from July 2016 to May 2022 were analyzed retrospectively. The inclusion criteria were as follows: (i) aged ≥18 years, (ii) received a standard 7+3 induction chemotherapy regimen or Venetoclax plus Hypomethylating Agents (including Azacitidine, Deoxycytidine) (VEN+HMA). All enrolled patients were diagnosed according to the WHO 2016 edition of myeloid neoplasms and acute leukemia[1]. All the samples were from Hematological Biobank, Jiangsu Biobank of Clinical Resources. This study was approved by the institutional review board or ethics committee of the First Affiliated Hospital of Soochow University and was conducted according to Declaration of Helsinki.

Molecular analyses

Genomic DNA was extracted using the Invitrogen DNA Extraction Kit from bone marrow-derived mononuclear cells samples obtained at initial diagnosis. Totally 51 frequently mutated genes in AML were detected in the cohort, which consist of a custom-designed 49-gene panel examined by next generation sequencing (NGS) (Ion S5™ System, Thermo Fisher, San Diego, CA, U.S.A.) and remaining genes (*CEBPA*, *FLT3-ITD*, *NPM1*) tested by Sanger sequencing. Potential germline *CEBPA* mutations were validated by matched remission samples, fingernails or buccal swab samples and excluded.

Statistical analyses

Categorical variables were compared by Chi-square test or Fisher's exact test. Continuous variables were examined through independent sample t-test or Wilcoxon rank-sum test between two groups and one-way analysis of variance (ANOVA) or Kruskal-Wallis H test among three groups. Pearson's correlation coefficient was used for correlation analysis. Overall survival (OS) was defined from the time of diagnosis to the time of death or last follow-up date. Relapse-free survival (RFS) was defined as the time from first complete remission (CR) or CR with incomplete hematologic recovery (CRi) to the date of disease relapse, death due to any cause or last follow-up. The Kaplan–Meier method and log-rank test were used to estimate and compare the OS and RFS. Landmark analysis was performed when the Kaplan-Meier curves for survival cross each other[2]. Univariate analysis and multivariate analysis were performed with cox proportional hazard model. Variables that were significant in the univariate analysis were included in the multivariable model. Propensity score

matching (PSM) was conducted by entering variables of white blood cell counts, cytogenetic risk category (favorable, intermediate, adverse), hematopoietic stem cell transplantation (HSCT) and induction treatment, ratio of 1:2, calipers of 0.02 between *CEBPA*^{mut} and *CEBPA*^{wt} groups. *P* value less than 0.05 was considered statistically significant. All statistical analyses were performed with R and SPSS (Version 20.0) software.

Table S1. Clinical characteristics of AML patients according to the status of *CEBPA* mutations.

Characteristic	***CEBPA***$^{\mathbf{wt}}$ n=813	***CEBPA***$^{\mathbf{bZIP\text{-}inf}}$ n=135	***CEBPA***$^{\mathbf{other\text{-}mut}}$ n=42	***P* value**
Age in years, median [IQR]	40.0 [30.0, 50.0]	36.0 [30.0, 45.5]	50.0 [36.3, 55.0]	<0.001
Sex (Male), n (%)	418 (51.4)	79 (58.5)	24 (57.1)	0.259
WBC count, x10^9/L	16.9 [4.5, 58.9]	16.3 [8.4, 68.7]	31.2 [5.1, 91.0]	0.150
Hemoglobin, g/L	84.0 [65.0, 104.0]	101.0 [82.0, 115.3]	88.0 [69.0, 107.5]	<0.001
Platelet count, x10^9/L	48.0 [25.0, 87.0]	28.0 [17.0, 49.0]	39.0 [25.0, 73.5]	<0.001
BM blasts (%)	55.0 [30.0, 76.0]	57.0 [38.0, 70.5]	65.0 [38.8, 79.9]	0.309
Available karyotype, n (%)	792 (97.4)	130 (96.3)	39 (92.9)	<0.001
favorable risk	232 (29.3)	0 (0.0)	2 (5.1)	
intermediate risk	423 (53.4)	127 (97.7)	34 (87.2)	
adverse risk	137 (17.3)	3 (2.3)	3 (7.7)	
Induction regimens, n (%)				0.381
7+3	686 (84.4)	120 (88.9)	35 (83.3)	
VEN+HMA	127 (15.6)	15 (11.1)	7 (16.7)	
Induction response, n (%)	800 (98.4)	134 (99.3)	42 (100)	<0.001
CR/CRi	450 (56.2)	118 (88.1)	26 (61.9)	
PR or NR	350 (43.8)	16 (11.9)	16 (38.1)	

Abbreviations: *AML* Acute myeloid leukemia, *WBC* white blood cell count, *BM* bone marrow, *VEN+HMA* Venetoclax plus hypomethylating agents, *CR/CRi* complete remission or CR with incomplete hematologic recovery, *PR* partial response, *NR* no response.

Table S2. Clinical features of *CEBPA*$^{bZIP-inf}$, *CEBPA*$^{other-mut}$ and *CEBPA*^{wt} AML patients after propensity score matching (PSM).

Characteristic	***CEBPA***^{wt} **n=304**	***CEBPA***$^{bZIP-inf}$ **n=130**	***CEBPA***$^{other-mut}$ **n=39**	***P* value**
Age in years, median [IQR]	49.0 [42.0, 55.0]	35.0 [30.0, 45.0]	48.0 [36.0, 55.5]	<0.001
Sex (Male), n (%)	144 (47.4)	77 (59.2)	22 (56.4)	0.062
WBC count, x10^9/L	16.9 [3.2, 59.2]	16.5 [8.5, 66.5]	29.3 [5.5, 72.6]	0.072
BM blasts (%)	58.0 [32.0, 76.0]	56.8[38.3, 70.8]	64.5 [39.5, 76.5]	0.666
Cytogenetics, n (%)				0.063
favorable risk	4 (1.3)	0 (0.0)	2 (5.1)	
intermediate risk	288 (94.7)	127 (97.7)	34 (87.2)	
adverse risk	12 (3.9)	3 (2.3)	3 (7.7)	
Induction regimens, n (%)				0.467
7+3	262 (86.2)	116 (89.2)	32 (82.1)	
VEN+HMA	42 (13.8)	14 (10.8)	7 (17.9)	
Induction response, n (%)				<0.001
CR/CRi	139(47.3)	113 (87.6)	25 (64.1)	
PR or NR	155 (52.7)	16 (12.4)	14 (35.9)	
HSCT, n (%)	171 (56.2)	75 (57.7)	22 (56.4)	0.962

Abbreviations: *WBC* white blood cell count, *BM* bone marrow, *VEN+HMA* Venetoclax plus hypomethylating agents, *HSCT* hematopoietic stem cell transplantation.

Table S3. Impact of clinical characteristics on OS and RFS by univariate and multivariate analysis.

	Univariate		Multivariate	
**OS**	**HR (95% CI)**	***P* value**	**HR (95% CI)**	***P* value**
Age	1.02 (1.02-1.05)	0.023	1.02 (1.00-1.04)	0.093
ASXL1^{mut}	2.66 (1.56-4.55)	<0.001	1.26 (0.58-2.72)	0.041
FLT3-ITD^{mut}	1.60 (1.13-2.24)	0.007	0.75 (0.48-1.18)	0.219
SF3B1^{mut}	4.61 (1.46-14.60)	0.009	4.58 (1.07-19.66)	0.041
CEBPA^{wt}				
CEBPA$^{bZIP\text{-}inf}$	0.14 (0.07-0.27)	<0.001	0.26 (0.12-0.57)	<0.001
CEBPA$^{other\text{-}mut}$	0.79 (0.46-1.38)	0.414	0.81 (0.44-1.50)	0.499
CR/CRi	0.34 (0.24-0.49)	<0.001	0.78 (0.51-1.21)	0.273
Relpase	10.4 (6.29-17.3)	<0.001	7.19 (4.33-11.92)	<0.001
HSCT in CR1 (time-dependent)	0.34 (0.22-0.52)	<0.001	0.58 (0.36-0.95)	0.031
**RFS**	**HR (95% CI)**	***P* value**	**HR (95% CI)**	***P* value**
Age	1.04 (1.02-1.05)	0.002	1.01 (0.99-1.03)	0.141
WBC ≥40(x10^9/L)	1.38 (0.99-1.94)	0.039	1.27 (0.88-1.84)	0.199
FLT3-ITD^{mut}	1.73 (1.23-2.42)	0.002	1.36 (0.93-2.00)	0.113
SF3B1^{mut}	4.28 (1.05-17.40)	0.040	1.52 (0.34-6.79)	0.581
KIT^{mut}	2.15 (1.00-4.60)	0.045	2.11 (0.97-4.60)	0.060
CEBPA^{wt}				
CEBPA$^{bZIP\text{-}inf}$	0.52 (0.35-0.79)	<0.001	0.61 (0.37-0.99)	0.047
CEBPA$^{other\text{-}mut}$	1.14 (0.67-1.95)	0.618	1.08 (0.61-1.89)	0.798
CR/CRi	0.63 (0.45-0.88)	0.007	0.72 (0.5-1.04)	0.083
HSCT in CR1 (time-dependent)	0.38 (0.26-0.56)	<0.001	0.34 (0.22-0.50)	<0.001

Abbreviations: *OS* overall survival, *mut* mutation, *wt* wild type, *RFS* relapse-free survival, *WBC* white blood cells, *CR/CRi* complete remission or CR with incomplete hematologic recovery, *HSCT* hematopoietic stem cell transplantation, *CR1* first complete remission.

Table S4. Prognostic factors for OS and RFS in *CEBPA*$^{bZIP-inf}$ AML patients.

	**Univariate**		**Multivariate**	
**OS**	**HR (95% CI)**	***P* value**	**HR (95% CI)**	***P* value**
KIT^{mut}	10.50 (2.15-51.30)	0.004	2.63 (0.30-23.04)	0.381
NRAS^{mut}	3.42 (0.85-13.70)	0.083	1.53 (0.27-8.82)	0.633
WT1^{mut}	3.16 (0.85-11.80)	0.087	2.21 (0.50-9.75)	0.296
Induction with 7+3				
VEN+HMA	9.30 (2.11-40.90)	0.003	5.14 (0.83-31.60)	0.078
**RFS**	**HR (95% CI)**	***P* value**	**HR (95% CI)**	***P* value**
WBC ≥40(x10^9/L)	1.97 (0.96-4.02)	0.064	1.95 (0.92-4.16)	0.082
KIT^{mut}	5.56 (1.65-18.70)	0.006	2.45 (0.63-9.55)	0.197
WT1^{mut}	1.99 (0.98-4.07)	0.058	2.24 (1.03-4.85)	0.042
NRAS^{mut}	2.16 (0.93-5.03)	0.073	3.14 (1.20-8.27)	0.020
Induction with 7+3				
VEN+HMA	4.73 (1.88-11.90)	<0.001	2.72 (1.01-7.30)	0.047
HSCT in CR1 (time-dependent)	0.08 (0.02-0.34)	<0.001	0.04 (0.01-0.19)	<0.001

Abbreviations: *OS* overall survival, *mut* mutation, *VEN+HMA* Venetoclax plus hypomethylating agents, *RFS* relapse-free survival, *WBC* white blood cells, *HSCT* hematopoietic stem cell transplantation, *CR1* first complete remission.

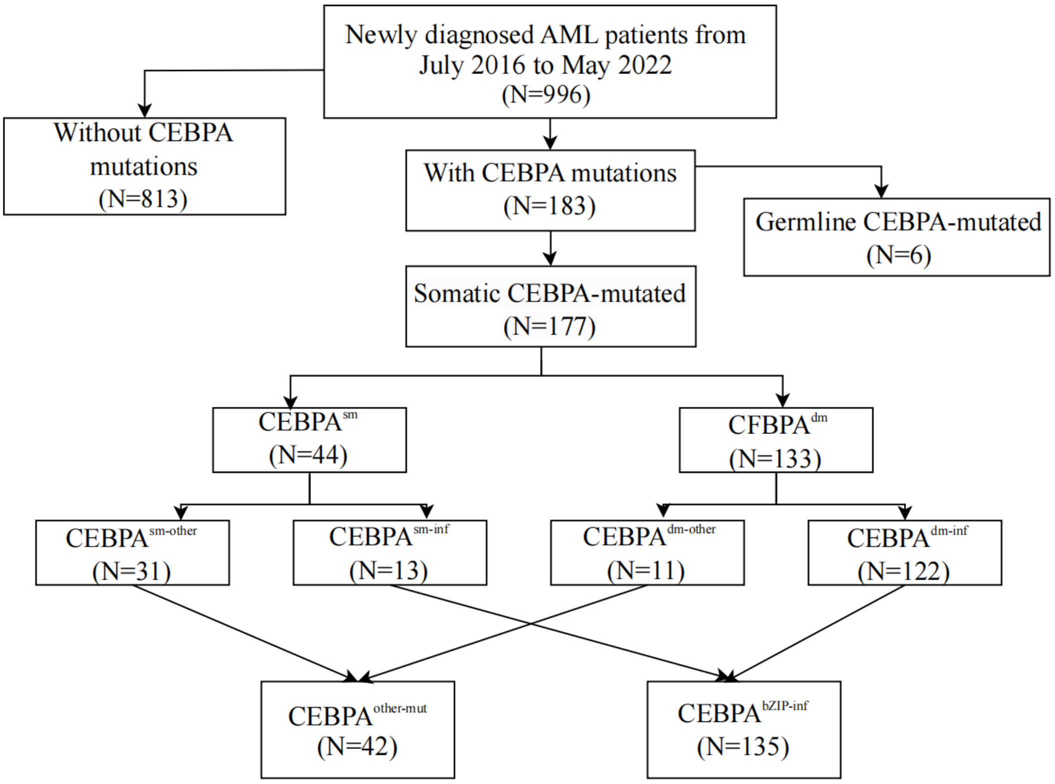

Figure S1. Algorithm for identifying *CEBPA* mutations in newly diagnosed adult AML patients. Abbreviations: *sm* single mutated, *dm* double mutated, *bZIP-inf* In-frame mutations within bZIP domain.

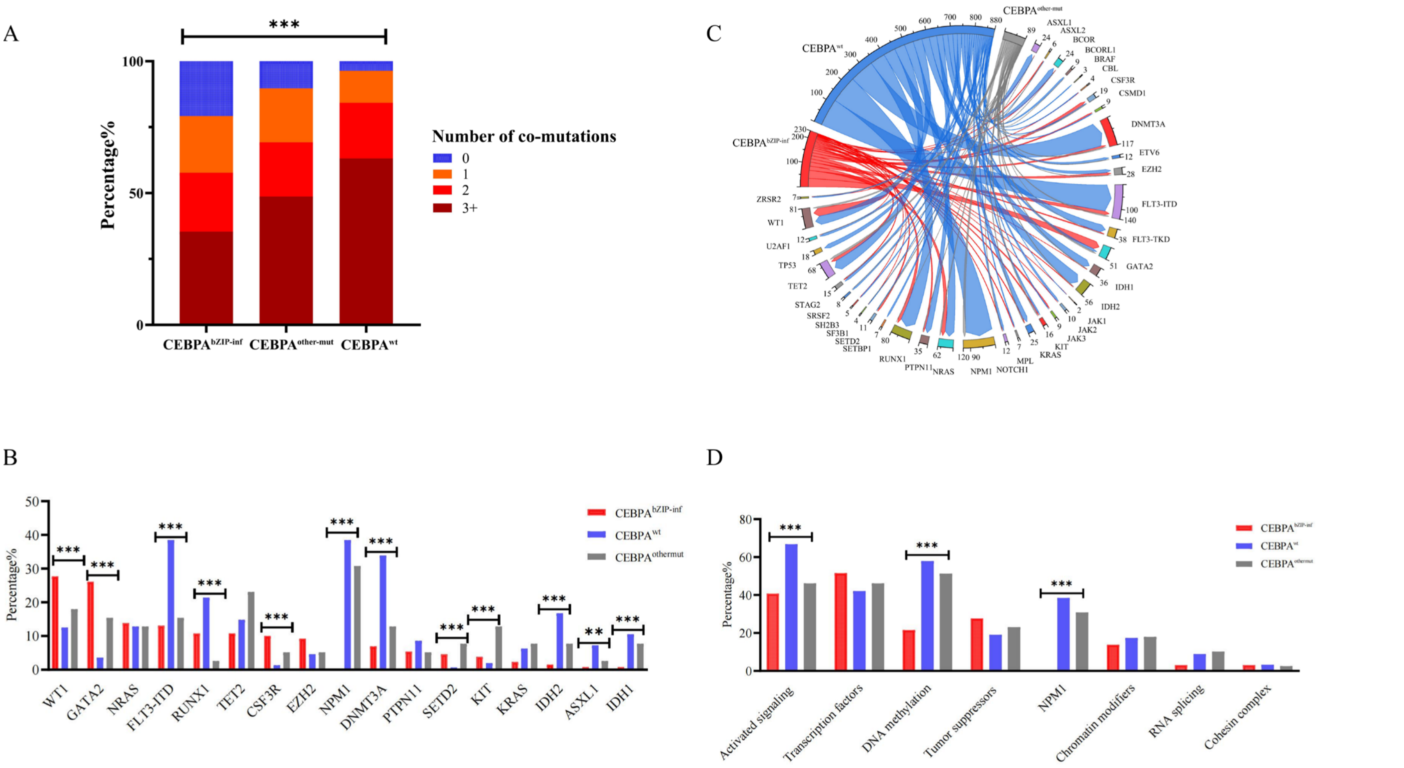

Figure S2. Molecular profiles of *CEBPA*$^{\text{bZIP-inf}}$, *CEBPA*$^{\text{other-mut}}$ and CEBPA^{wt} patients as determined by gene sequencing. **A.** Number of concomitant mutations in CEBPA$^{\text{bZIP-inf}}$**,** *CEBPA*$^{\text{other-mut}}$ and *CEBPA*^{wt} patients. **B.** Frequency distribution of additional gene mutations identified in three groups. **C.** Circos diagram depicting the relative frequency and pairwise co-occurrence of mutations according to *CEBPA* mutations status. **D.** Functional classifications of concomitant mutations within three groups.

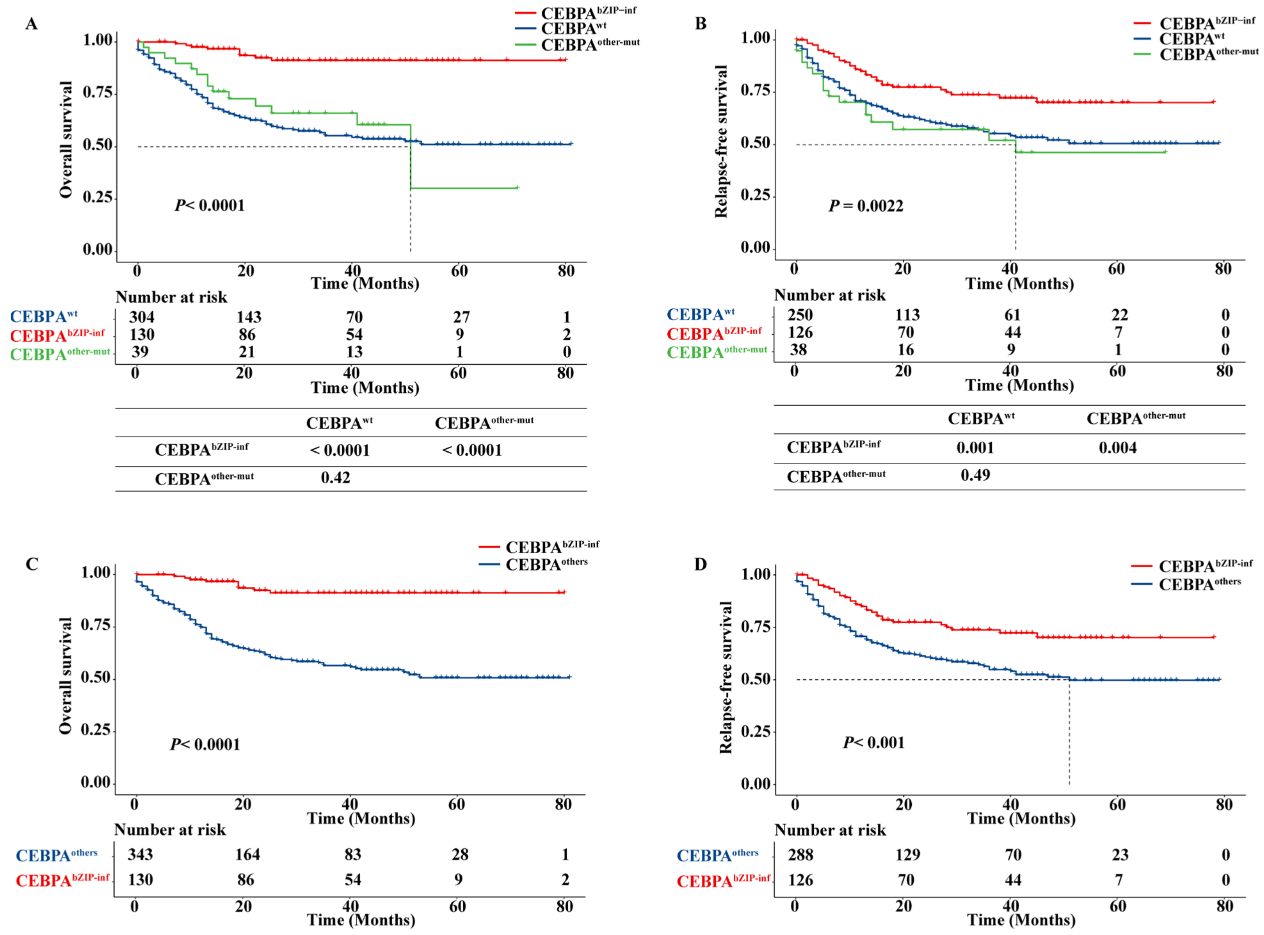

Figure S3. Survival analysis according to *CEBPA* mutation status. **A-B** showed OS and RFS of *CEBPA*$^{\text{bZIP-inf}}$, *CEBPA*$^{\text{other-mut}}$ and *CEBPA*^{wt} patients*;* Results of log-rank tests from pairwise comparisons are provided below the x-axis. **C-D** presented OS and RFS of *CEBPA*$^{\text{bZIP-inf}}$ and *CEBPA*^{others} cases. Abbreviations: *CEBPA*^{others} group including *CEBPA*$^{\text{other-mut}}$ and *CEBPA*^{wt} groups.

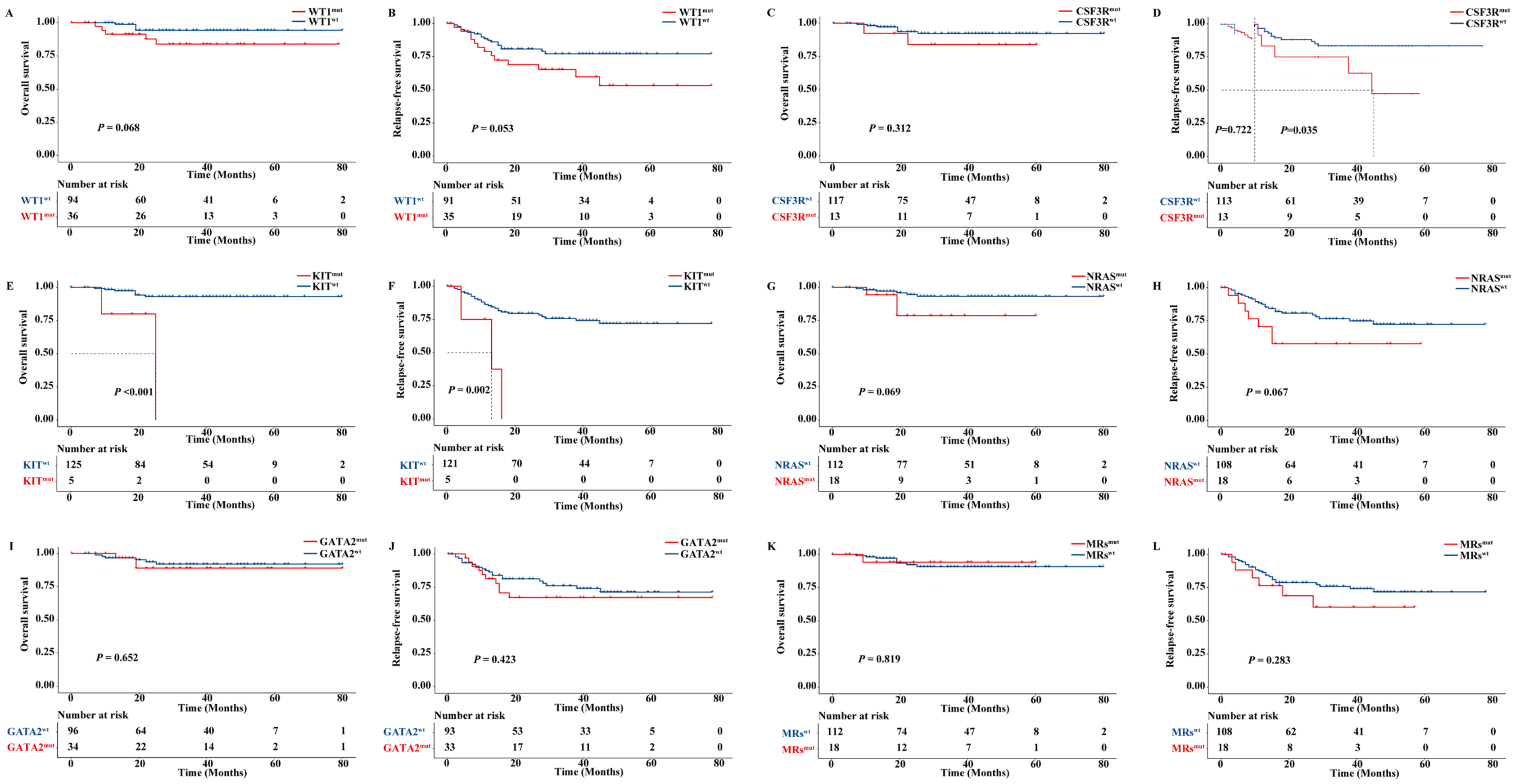

Figure S4. Kaplan-Meier Survival Curves for OS and RFS in *CEBPA*$^{\text{bZIP-inf}}$ patients according to the status of different concomitant mutations. **A-B.** Wilms Tumour 1 (*WT1*) mutation status; **C-D.** Colony-Stimulating Factor 3 Receptor (*CSF3R*); **E-F.** KIT proto-oncogene, receptor tyrosine kinase (*KIT*); **G-H**. neuroblastoma RAS viral oncogene homolog (*NRAS*); **I-J.** GATA binding protein 2 (*GATA2*); **K-L.** myelodysplasia-related mutations (MRs). Abbreviations: *mut* mutant, *wt* wild type.

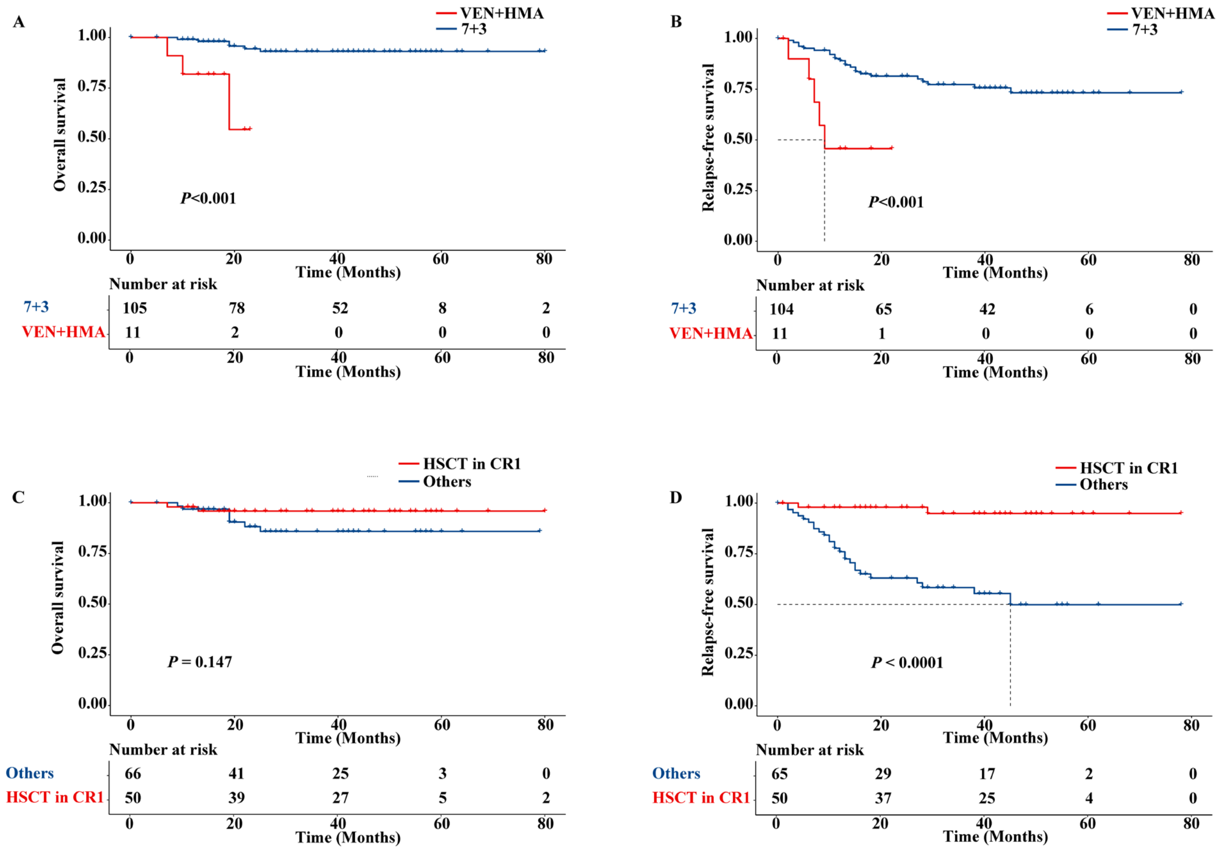

Figure S5. Kaplan-Meier Survival Curves for OS and RFS in *CEBPA* bZIP^{InDel} patients. **A-B** compared survival outcomes among patients treated with VEN+HMA or standard 7+3; **C-D** presented survival outcomes by consolidation (HSCT in CR1 or not). Abbreviations: *bZIP*^{InDel} in-frame insertions/deletions within bZIP domain.

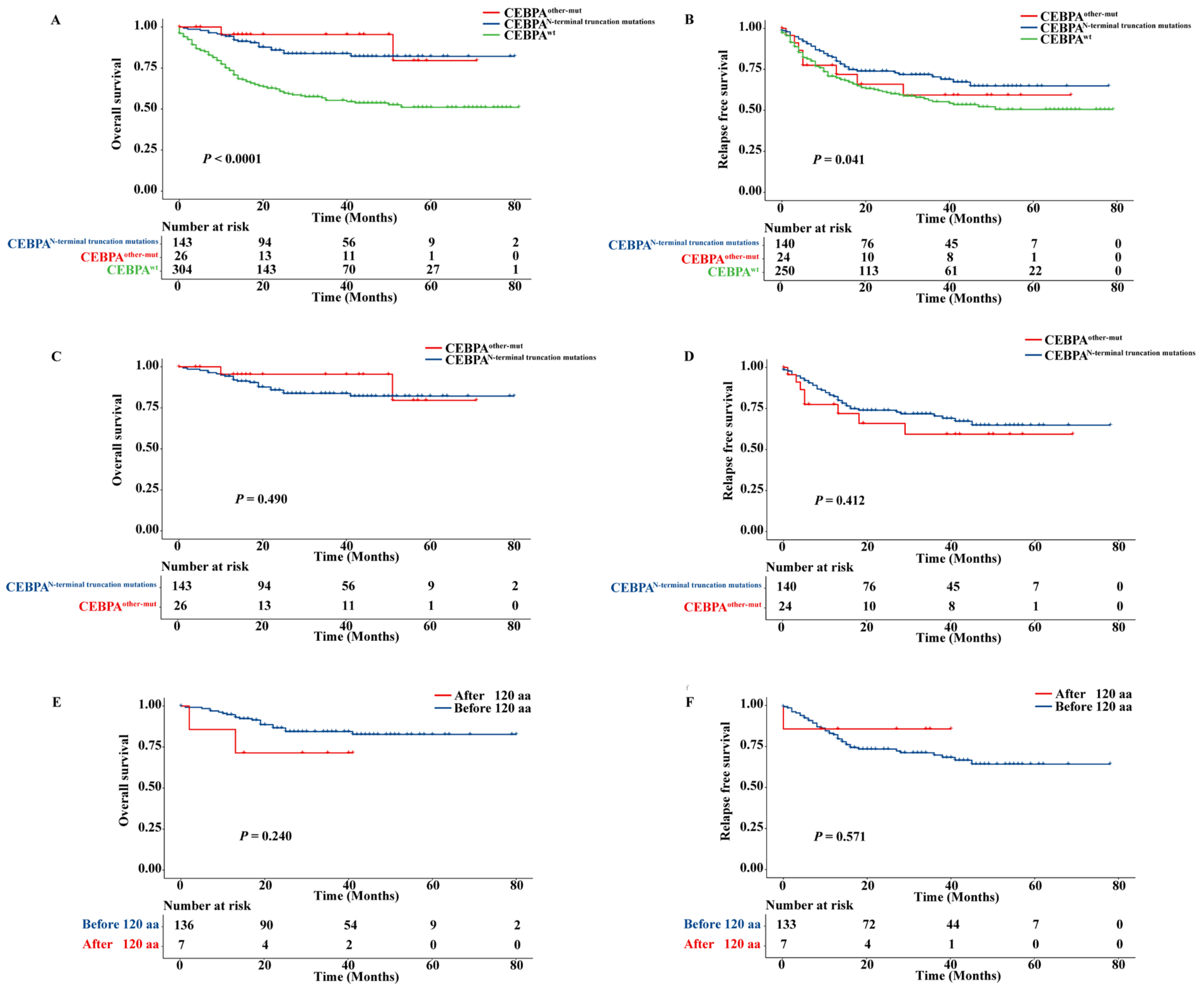

Figure S6. Kaplan-Meier plots of OS and RFS in *CEBPA* mutated AML patients according to the localization and type of the mutation. **A-D**. Kaplan-Meier Survival Curves for *CEBPA* N-terminal truncation mutated patients and those with other *CEBPA* mutations. **E-F.** Kaplan-Meier Curves assessing OS and RFS in patients with N-terminal truncated mutations before and after 120 amino acid. Abbreviations: *aa* amino acid.

References

1. Arber DA, Orazi A, Hasserjian R, Thiele J, Borowitz MJ, Le Beau MM, et al. The 2016 revision to the World Health Organization classification of myeloid neoplasms and acute leukemia. Blood. 2016;127:2391-405.

2. Zhao F, Wang J, Chen M, Chen D, Ye S, Li X, et al. Sites of synchronous distant metastases and prognosis in prostate cancer patients with bone metastases at initial diagnosis: a population-based study of 16,643 patients. Clin Transl Med. 2019;8:30.
